# The Identification of Potential Drugs for Dengue Hemorrhagic Fever: Network-Based Drug Reprofiling Study

**DOI:** 10.2196/37306

**Published:** 2023-05-09

**Authors:** Praveenkumar Kochuthakidiyel Suresh, Gnanasoundari Sekar, Kavya Mallady, Wan Suriana Wan Ab Rahman, Wan Nazatul Shima Shahidan, Gokulakannan Venkatesan

**Affiliations:** 1 Central Research Facility Sri Ramachandra Institute of Higher Education and Research Chennai India; 2 Department of Biotechnology Bharathidasan Institute of Technology Campus Anna University Tiruchirappalli India; 3 Centre for Toxicology and Developmental Research Sri Ramachandra Institute of Higher Education and Research Chennai India; 4 School of Dental Sciences Universiti Sains Malaysia Health Campus Kelantan Malaysia

**Keywords:** dengue hemorrhagic fever, drug reprofiling, network pharmacology, network medicine, DHF, repurposable drugs, viral fevers, drug repurposing

## Abstract

**Background:**

Dengue fever can progress to dengue hemorrhagic fever (DHF), a more serious and occasionally fatal form of the disease. Indicators of serious disease arise about the time the fever begins to reduce (typically 3 to 7 days following symptom onset). There are currently no effective antivirals available. Drug repurposing is an emerging drug discovery process for rapidly developing effective DHF therapies. Through network pharmacology modeling, several US Food and Drug Administration (FDA)-approved medications have already been researched for various viral outbreaks.

**Objective:**

We aimed to identify potentially repurposable drugs for DHF among existing FDA-approved drugs for viral attacks, symptoms of viral fevers, and DHF.

**Methods:**

Using target identification databases (GeneCards and DrugBank), we identified human–DHF virus interacting genes and drug targets against these genes. We determined hub genes and potential drugs with a network-based analysis. We performed functional enrichment and network analyses to identify pathways, protein-protein interactions, tissues where the gene expression was high, and disease-gene associations.

**Results:**

Analyzing virus-host interactions and therapeutic targets in the human genome network revealed 45 repurposable medicines. Hub network analysis of host-virus-drug associations suggested that aspirin, captopril, and rilonacept might efficiently treat DHF. Gene enrichment analysis supported these findings. According to a Mayo Clinic report, using aspirin in the treatment of dengue fever may increase the risk of bleeding complications, but several studies from around the world suggest that thrombosis is associated with DHF. The human interactome contains the genes *prostaglandin-endoperoxide synthase 2* (*PTGS2*), *angiotensin converting enzyme (ACE)*, and *coagulation factor II, thrombin* (*F2)*, which have been documented to have a role in the pathogenesis of disease progression in DHF, and our analysis of most of the drugs targeting these genes showed that the hub gene module (human-virus-drug) was highly enriched in tissues associated with the immune system (*P*=7.29 × 10^–24^) and human umbilical vein endothelial cells (*P*=1.83 × 10^–20^); this group of tissues acts as an anticoagulant barrier between the vessel walls and blood. Kegg analysis showed an association with genes linked to cancer (*P*=1.13 × 10^–14^) and the advanced glycation end products–receptor for advanced glycation end products signaling pathway in diabetic complications (*P*=3.52 × 10^–14^), which indicates that DHF patients with diabetes and cancer are at risk of higher pathogenicity. Thus, gene-targeting medications may play a significant part in limiting or worsening the condition of DHF patients.

**Conclusions:**

Aspirin is not usually prescribed for dengue fever because of bleeding complications, but it has been reported that using aspirin in lower doses is beneficial in the management of diseases with thrombosis. Drug repurposing is an emerging field in which clinical validation and dosage identification are required before the drug is prescribed. Further retrospective and collaborative international trials are essential for understanding the pathogenesis of this condition.

## Introduction

Dengue fever, also known as “breakbone fever,” is characterized by acute, severe fever in patients 3 to 14 days after they are bitten by an infected mosquito. Migraine, retro-orbital pain, myalgia, muscle ache, signs of hemolytic anemia, rash, and a low white blood cell count are only a few of the symptoms [1]. Dengue hemorrhagic fever (DHF), a severe and sometimes fatal manifestation of the disease, affects certain patients with dengue fever. These patients may show warning signs of serious disease close to the period the fever begins to diminish (typically 3 to 7 days following symptom onset). Severe abdominal discomfort, continuous vomiting, a significant change in temperature (from fever to hypothermia), hemorrhagic manifestations, or a change in mental status (eg, irritability, confusion, or obtundation) are also warning indicators [2]. Restlessness, chilly, clammy skin, a rapid, weak pulse, and a narrowing of pulse pressure (both systolic blood pressure and diastolic blood pressure) are all early indications of shock. Patients with dengue fever should be advised to return to the hospital if any of these symptoms appear.

According to one estimate, 390 million dengue virus infections occur each year (95% credible interval [CI] 284 million to 528 million), with 96 million (95% CI 67 million to 136 million) showing clinical symptoms of any severity [3]. According to the World Health Organization (WHO), 3.9 billion individuals are at risk of contracting the dengue virus. Although there is a risk of infection in 129 nations, Asia bears 70% of the actual burden. Over the last 2 decades, the number of dengue cases reported to the WHO has increased more than 8 times, from 505,430 cases in 2000 to over 2.4 million in 2010 and 5.2 million in 2019. Between 2000 and 2015, the number of deaths reported grew from 960 to 2032. This worrying rise in case numbers can be explained in part by a shift in national practices for recording and reporting dengue fever to health ministries and the WHO. However, it also symbolizes the government’s acknowledgment of the problem, and hence the need to disclose the prevalence of dengue fever [4]. As a result, while the complete global burden of the disease remains unknown, the observed growth only takes us closer to a more precise estimate of the full extent of the burden.

In 2021, dengue fever increased in Bangladesh, Brazil, the Cook Islands, Ecuador, India, Indonesia, the Maldives, Mauritania, Mayotte (an overseas department of France), Nepal, Singapore, Sri Lanka, Sudan, Thailand, Timor-Leste, and Yemen. Dengue fever was also still a problem in Brazil, the Cook Islands, Colombia, Fiji, Kenya, Paraguay, Peru, and Reunion Island in 2021 [5]. The COVID-19 epidemic is placing an enormous strain on health care and management systems all across the world. During this critical period, the WHO has stressed the significance of maintaining efforts to prevent, identify, and treat vector-borne diseases such as dengue fever and other arboviral infections as case numbers rise in various countries, putting urban people at risk for both diseases [6].

Recent systems biology developments suggest a unique testable hypothesis for systematic drug repurposing [7,8]. This can greatly decrease the time spent on research and development compared to traditional drug development programs. The typical strategy takes 10 to 16 years to develop a new treatment. A medication repurposing plan costs $1.6 billion to create, whereas a typical strategy costs $12 billion [9]. The identification of new targets and illness proteins has been made possible by rapid advances in genomic, proteomic, structural, functional, and systems investigations of existing targets and other disease proteins [10].

In this study, we provide an embedded medicine platform that uses a network-based method to quantify the association of DHF with human-host interactions, we examine the efficacy of existing US Food and Drug Administration (FDA)-approved medications as potentially repurposable drugs, and we also examine their associations with DHF-host genes. To discover and prioritize existing pharmacological targets in the DHF pathway, we chose FDA-approved medications from a clinical registry database.

## Methods

### Building the DHF-Human Interactome

We performed an extensive electronic-literature similarity search from January 2000 to January 2022 for keywords related to DHF and human interactions, including “dengue hemorrhagic fever,” “dengue hemorrhagic fever and human gene interaction,” and “dengue hemorrhagic fever human interactome,” with a focus on reviews, editorials, commentary, letters, case reports, and original research manuscripts published on PubMed, Google Scholar, and other widely used databases. We manually removed duplicates based on variables such as author, nationality, and collaborations, and we finally extracted 31 articles. We performed a database similarity search using GeneCards and found 588 DHF-targeting human genes. A total of 59 host-interacting DHF genes were sorted based on a hit score of 50. Experimental evidence for interactions between human proteins and dengue virus proteins was obtained with high-throughput yeast 2-hybrid screening methods [11]. Recently, Dey and Mukhopadhyay [12] reported the development of DenvInt, a database of manually curated experimental data of dengue protein and host protein interactions. We merged the data from published references to DenvInt and used them in our analysis along with the dengue-host interactome data from recent investigations. Infectious diseases are the result of molecular crosstalk between hosts and their pathogens. This crosstalk is in part mediated by host-pathogen (HP) protein-protein interactions (PPIs). HP-PPIs play crucial roles in infections [13]. The best way of unveiling their mechanisms is to investigate the HP-PPI network [14].

### Human (Host) Gene–DHF Gene Interactome

The key host genes involved in DHF were identified from the GeneCards database using the search terms “dengue hemorrhagic fever” and “dengue hemorrhagic fever interacting human genes.” GeneCards is a searchable, integrative database that provides comprehensive, user-friendly information on all annotated and predicted human genes. The knowledge base automatically integrates gene-centric data from approximately 150 web sources, including genomic, transcriptomic, proteomic, genetic, clinical, and functional information. As of January 13, 2022, GeneCards comprises 326,787 genes, including 18,870 disease genes and 500 host genes [15]. The functional genes identified from GeneCards and related literature were collected and are presented in Multimedia Appendix 1. The PPI network was built with Cytoscape (version 3.9.0; Cytoscape Consortium) and Gephi (version 0.9.2; Gephi Association) [16].

### Drug Target (Human Gene) Interactome

We collected 87 FDA-approved antiviral and 137 anti-DHF drugs from the Therapeutic Target Database, compared them with the results from the DrugBank database [17,18], identified drug targets, and formulated them as a data set (Multimedia Appendix 1). Human-drug interactions are based on drug targets (ie, drug targeting genes); these were visualized using Cytoscape [19]. The nodes in a network represent antiviral drugs or anti-DHF drugs and the edges of the network represent drugs targeting human genes [20].

### Building the Drug-to-Human Interactome

A network pharmacology–based host–DHF–antiviral–anti-DHF drug interactome was constructed by assembling the host-DHF interacting proteins with or without antivirals and anti-DHF drugs. The PPI network was built with Gephi [21] and Cytoscape. Each node in the constructed PPI network indicates a host gene and an edge indicates an interacting drug target.

### Network Hub Gene Identification

Highly connected nodes (hubs) in biological networks are topologically important to the structure of the network and have also been shown to be preferentially associated with a range of phenotypes of interest. Hub genes can be identified using the Contextual Hub Analysis Tool plug-in of Cytoscape [22], which enables users to easily construct and visualize a network of interactions from a gene list of interest.

### Functional Enrichment Analysis of Genes and Drugs

Functional enrichment analysis is a method to determine classes of genes or drugs that are overrepresented in a large group of genes or drugs and may have relations with disease phenotypes. This approach uses statistical methods to determine significantly enriched groups of genes. The biological relevance and functional pathways of our data sets were revealed by enriching the semantic similarities of the pathways and tissue. All functional enrichment analyses were performed using the Enrichr enrichment platform (Icahn School of Medicine) [23] as additional evidence for drug repurposing. Enrichr is a comprehensive gene enrichment analysis platform that comprises 382,208 terms from 192 libraries.

## Results

### Human (Host)-DHF (Viral) Gene Interactome

We constructed a host-DHF interactome consisting of 59 interacting genes with 60 nodes and 59 edges (Multimedia Appendix 2, Figure S1A). A Kegg pathway enrichment analysis indicated that genes involved in the advanced glycation end products–receptor for advanced glycation end products (AGE-RAGE) signaling pathway in diabetic complications were most enriched (*P*=3.01^32^), which indicates that patients with DHF have a higher chance of poor blood sugar management; meanwhile, AGE-RAGE signaling has been shown to increase oxidative stress and promote diabetes-mediated vascular calcification through activation of nicotinamide adenine dinucleotide phosphate oxidase-1 and decreased expression of superoxide dismutase type 1 [24]. Chagas disease (*P*=4.65 × 10^–32^) and influenza A (*P*=6.17 × 10^–31^) pathways were also typically enriched. Compared to the Kegg pathway analysis, a reactome pathway analysis indicated that the immune system (*P*=3.93 × 10^–28^) and cytokine signaling in the immune system (*P*=7.06 × 10^–27^) were enriched, which indicates that DHF most strongly hijacks the human immune system–associated gene pathways; a gene set was also identified in which the immune system (*P*=1.12 × 10^–61^) and bronchoalveolar lavage (*P*=2.85 × 10^–50^) tissues were most enriched (Multimedia Appendix 3, Figure S1B).

### Host–Viral–Antiviral Drug Target Interactome

A host–DHF–antiviral drug interactome was built with 298 nodes and 370 edges from 237 interacting genes (Multimedia Appendix 4, Figure S2A). Kegg pathway gene enrichment analysis revealed that upon antiviral drug administration, the most enriched gene was a neuroactive ligand–receptor interaction (*P*=3.22 × 10^–45^), that is, a collection of genes associated with intracellular and extracellular signaling pathways in the plasma membrane and mitogen-activated protein kinase pathways (*P*=9.57 × 10^–45^), which relay, amplify, and integrate signals from a diverse range of stimuli and elicit appropriate physiological responses in mammalian cells, including cellular proliferation, differentiation, and development; an inflammatory response; and apoptosis (Multimedia Appendix 5, Figure S2B). The reactome pathway analysis indicated that pathway genes were enriched in phase 2, the plateau phase (*P*=2.85 × 10^–34^), which sustains cardiac action potential muscle contraction [25,26] and transmission across chemical synapses (ie, neurotransmitters; *P*=6.03 × 10^–34^) after antiviral drug administration for DHF, as were genes in adult (*P*=2.52 × 10^–69^) and immune-system (*P*=1.77 × 10^–49^) tissue types.

### Host–Viral–Anti-DHF Drug Target Interactome

A host–DHF interactome–anti-DHF drug interactome was built with 558 nodes and 861 edges from 419 interacting genes (Multimedia Appendix 6, Figure S3A). Neuroactive ligand–receptor interaction (*P*=3.37 × 10^–75^) and the cAMP signaling pathway (*P*=2.29 × 10^–54^), which is also known as the adenylyl cyclase pathway and is a G protein–coupled receptor-triggered signaling cascade used in cell communication, were the most enriched gene pathways according to Kegg pathway analysis (Multimedia Appendix 7, Figure S3B). Amine ligand–binding receptors (*P*=1.76 × 10^–47^), which act as neurotransmitters in humans, and signal transduction (*P*=1.40 × 10^–46^), which involves the binding of extracellular signaling molecules and ligands to receptors located on the cell surface, were highly enriched. Adult (*P*=1.74 × 10^–59^) and immune-system (*P*=3.35 × 10^–54^) tissue types were the most prominent after anti-DHF drug administration in COVID-19 patients.

### Host–Viral–Antiviral–Anti-DHF Drug Target Interactome

**Figure 1:**
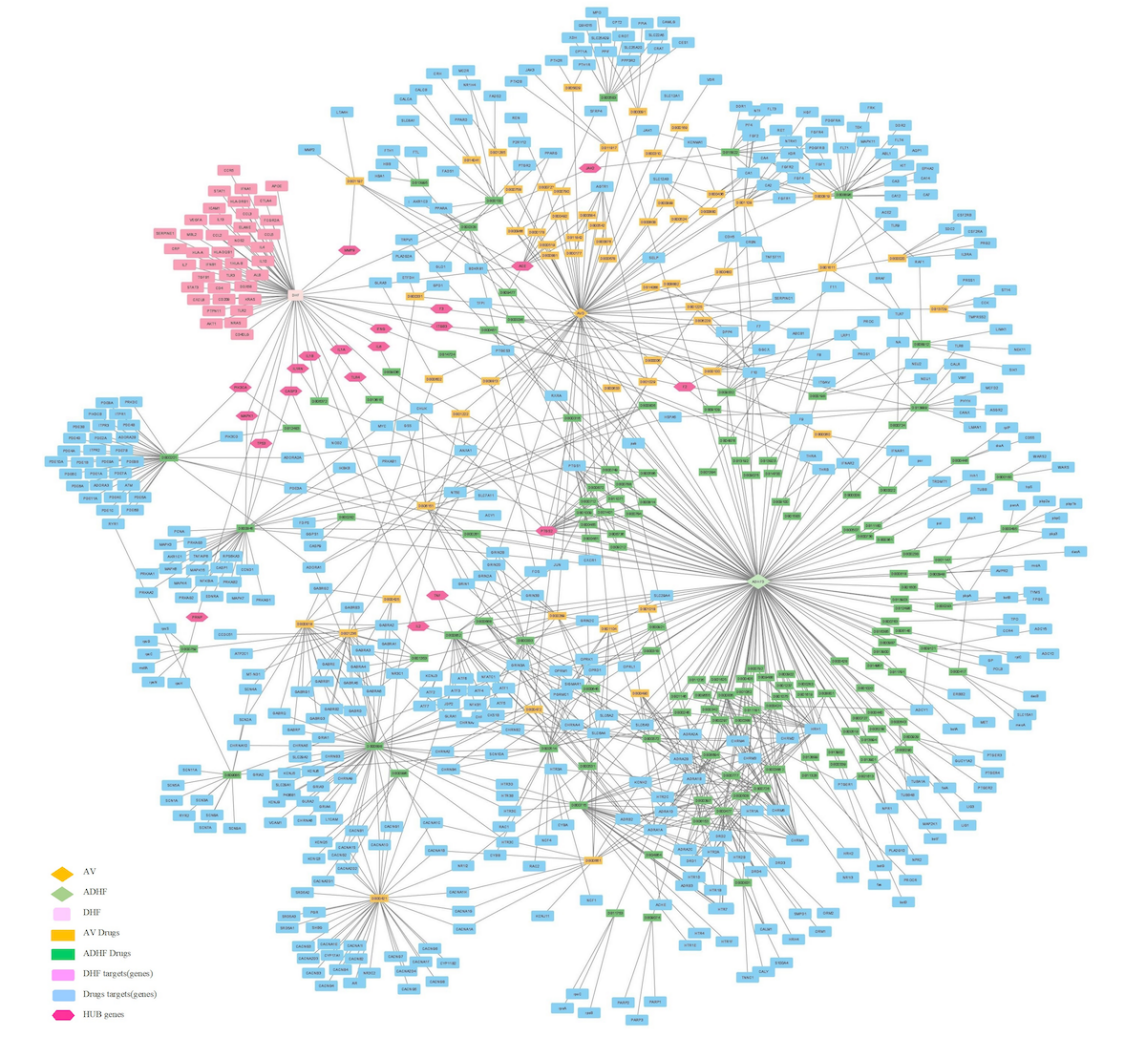
Human-anti–dengue hemorrhagic fever–antiviral drug–anti–dengue hemorrhagic fever interaction network. ADHF: anti–dengue hemorrhagic fever; AV: antiviral; DB: DrugBank; DHF: dengue hemorrhagic fever. Higher-resolution version of this figure is available in Multimedia Appendix 8.

**Figure 2:**
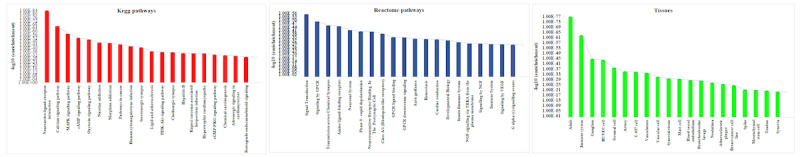
Enrichment analysis of human-anti–dengue hemorrhagic fever–antiviral drug–anti–dengue hemorrhagic fever. (A) Kegg pathways, (B), reactome pathways, and (C) tissues. Higher-resolution version of this figure is available in Multimedia Appendix 9.

Based on all the interactomic data sets, we combined all the data sets to frame a network-based drug reprofiling approach to testing their robustness, which involved a network with 717 nodes and 1175 edges from 487 interacting genes (Figure 1). Gene functional enrichment analysis of the Kegg pathway revealed that gene sets involved in neurotransmitter pathways (*P*=1.13 × 10^–84^) and calcium-signaling pathways (*P*=1.78 × 10^–66^) were highly enriched, similarly to previous drug-related host-virus interactomes in humans; these also provide a stable outcome when combined drug administration (eg, an antiviral and an anti-DHF drug) is used for patients with systemic DHF (Figure 2). The majority of the gene set was enriched in adult (*P*=1.53 × 10^–77^) and immune-system (*P*=3.07 × 10^–63^) tissues. Genes related to signal transduction (*P*=6.76 × 10^–56^) and signaling by G protein–coupled receptors (*P*=1.96 × 10^–49^) were the prevalent reactome pathways enriched in the patients with DHF and combined drug administration.

### Network-Based Drug Repurposing Based on Hub Gene Analysis

**Figure 3:**
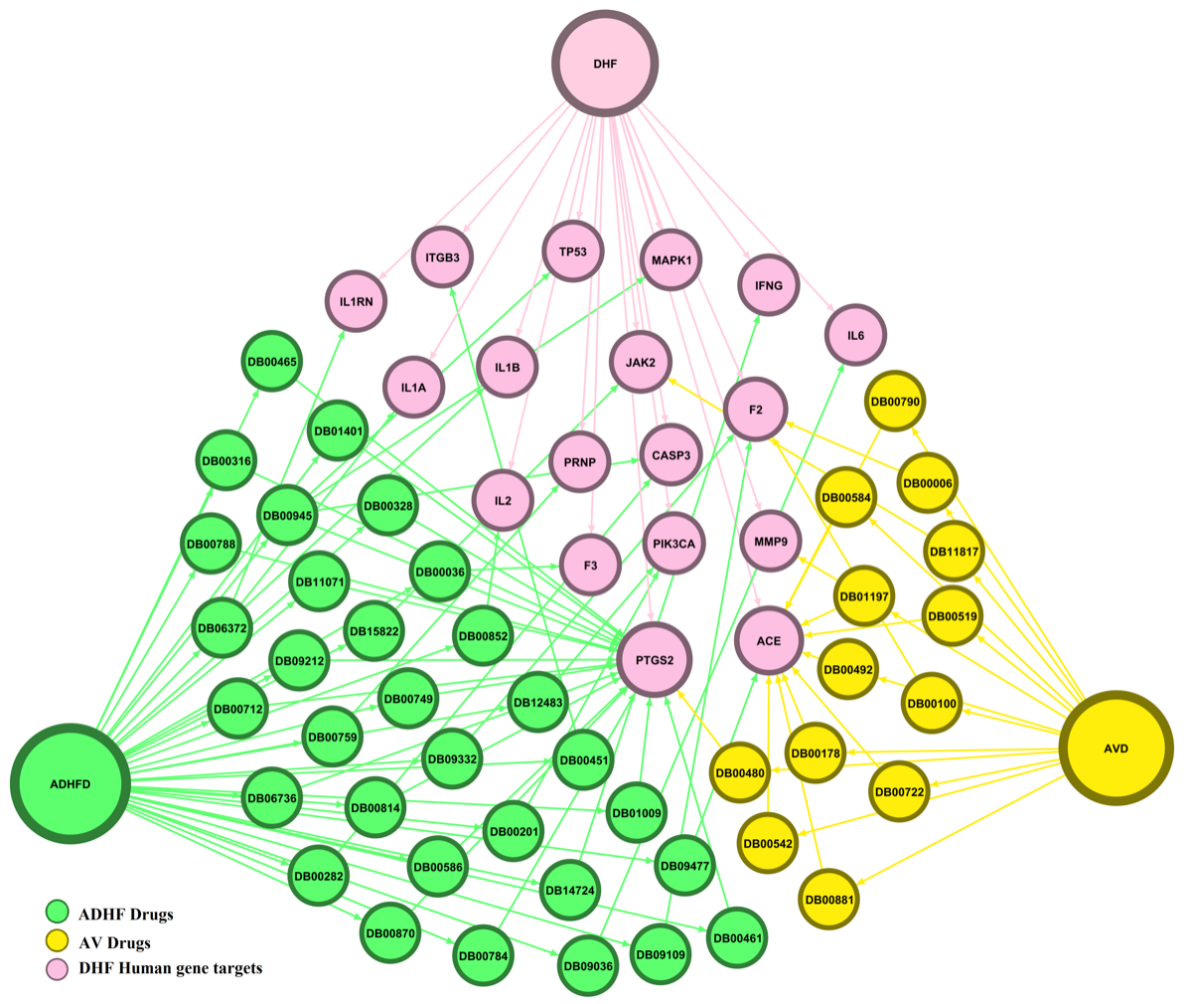
Representation of human-interacting anti-dengue hemorrhagic fever–antiviral drug–anti-dengue hemorrhagic fever hub network. ADHFD: anti–dengue hemorrhagic fever drugs; AVD: antiviral drugs; DB: DrugBank; DHF: dengue hemorrhagic fever.

**Figure 4:**
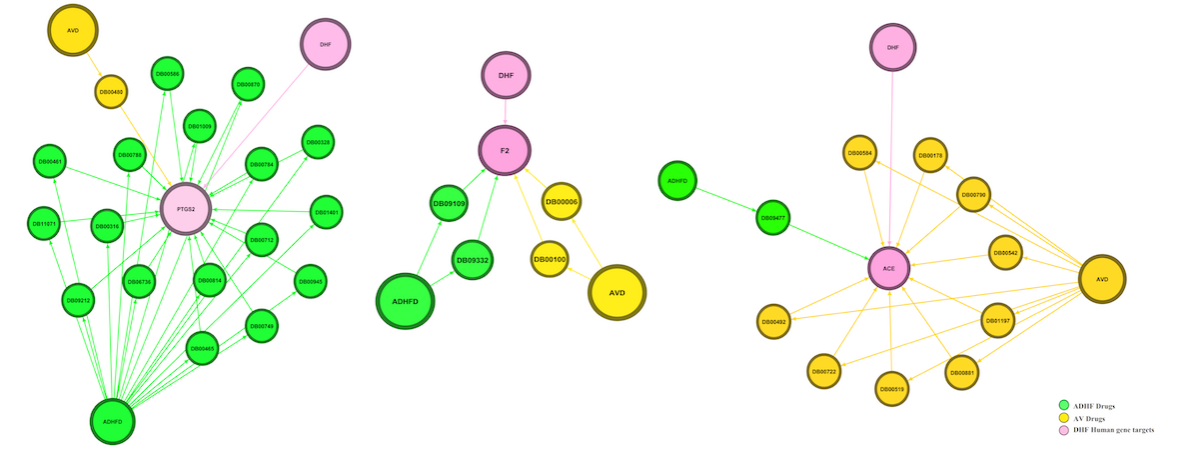
Gene interactions in hub network for (A) *PTGS2*, (B), *F2*, and (C) *ACE*. ADHFD: anti–dengue hemorrhagic fever drugs; AVD: antiviral drugs; DB: DrugBank; DHF: dengue hemorrhagic fever.

**Figure 5:**
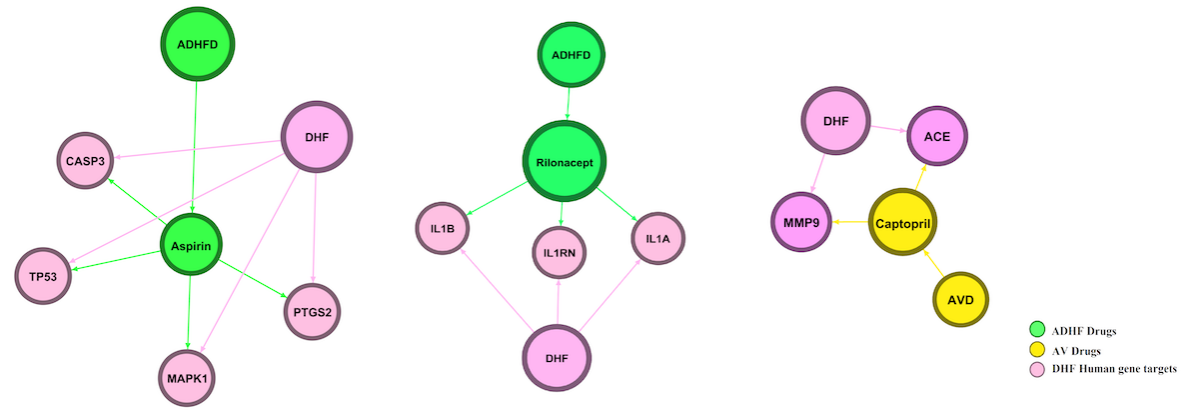
Repurposable drugs identified through hub network analysis: (A) aspirin, (B) rilonacept, and (C) capropril. ADHFD: anti–dengue hemorrhagic fever drugs; AVD: antiviral drugs; DHF: dengue hemorrhagic fever.

We predicted a hub gene module containing 20 interacting genes (66 nodes and 113 edges) from the above interactome of the host-virus-drugs systems framework (Figure 3). A total of 45 drugs were repurposed from the hub gene module, of which 13 were antiviral drugs and 32 were anti-DHF drugs. From the hub gene–drug association network, we determined that 3 major drugs bound efficiently with DHF-targeting human genes: aspirin, captopril, and rilonacept. Thus, these are efficient FDA-approved drugs that can be used in the treatment of DHF (Figure 4). We identified 18 *PTGS2* genes, 10 *ACE* genes, and 4 *F2* genes targeting drugs in hub genes in the network (Figure 2A). Interestingly, 18 of 17 *PTGS2*-targeting drugs were anti-DHF drugs and 10 of 9 *ACE*-targeting genes were antiviral drugs. Moreover, *F2*-targeting drugs had equal numbers of these 2 types of drugs: they included 2 antiviral drugs and 2 anti-DHF drugs (Figure 5).

**Figure 6:**
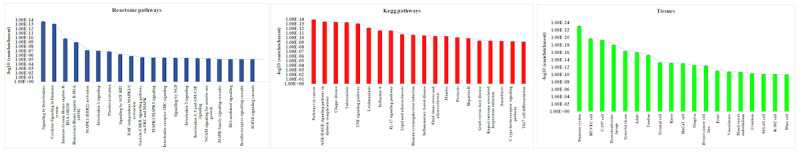
Functional hub gene enrichment analyses: (A) reactome pathways, (B), Kegg pathways, and (C) tissues. Higher-resolution version of this figure is available in Multimedia Appendix 10.

Gene enrichment analysis showed that the hub gene module was highly enriched in tissues associated with the immune system (*P*=7.29 × 10^–24^) and human umbilical vein endothelial cells (*P*=1.83 × 10^–20^). This group of tissues acts as an anticoagulant barrier between the vessel walls and blood. Kegg analysis showed that genes associated with cancer (*P*=1.13 × 10^–14^) and the AGE-RAGE signaling pathway in diabetic complications (*P*=3.52 × 10^–14^) were enriched, which indicates that DHF patients with diabetes and cancer are at risk of higher pathogenicity. Reactome pathway gene enrichment analysis provided evidence that immune system–associated pathways, including signaling by interleukins (*P*=2.04^–14^) and cytokine signaling in the immune system (*P*=7.12^–14^), were most enriched (Figure 6).

### Functional Enrichment Analysis of Drugs Based on Hub Gene Prediction

**Figure 7:**
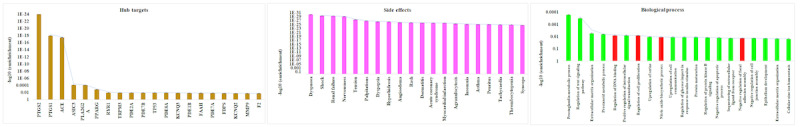
Functional hub gene–drug enrichment analyses: (A) hub targets, (B) side effects, and (C) biological process. Higher-resolution version of this figure is available in Multimedia Appendix 11.

Hub gene analysis showed a total of 45 repurposable drugs in which the human pathogen gene interacted with the drug target. The hub gene mechanism also showed where the genes were expressed in biological systems and side effects (Figure 7). Flurbiprofen, mefenamic acid, acetylsalicylic acid, indomethacin, naproxen, ketoprofen, acetaminophen, ketorolac, aceclofenac, lenalidomide, diclofenac, suprofen, loxoprofen, and nabumetone targeted the hub gene *PTGS2*. Module dyspnea, shock, renal failure, nervousness, and tension were prominent side effects of these drugs. The prostaglandin metabolic process (*P*=.00016) and regulation of the Wingless-related integration site signaling pathway (*P*=.00031 were prominent upregulated gene expression pathways after administration of the above drugs.

## Discussion

### Principal Findings

We systematically studied the association of dengue viral interactions with the human genome through network-based association analysis. We hypothesized that a host protein that is functionally associated with this virus is localized in a corresponding subnetwork within the comprehensive human interactome network. The host dependency factors mediating virus infection and effective molecular targets should be identified for developing broad-spectrum antiviral drugs and anti-DHF drugs. In our network-based analysis, we identified 45 repurposable drug candidates against DHF, including 13 antivirals targeting human genes and 32 anti-DHF drugs targeting 20 human genes. The most prevalent side effects identified in repurposed drug enrichment were dyspnea and shock. The *PTGS2*, *F2*, and *ACE* genes were highly targeted by the repurposed drugs.

### Comparison to Prior Work

The pathogenicity of the *PTGS2* and *COX-2* gene pathways in the progression of DHF has already been reported [27]. Most importantly, the *PTGS2* gene has a direct relationship with severe dengue, in which the blood vessels become damaged and leaky, and the number of clot-forming cells (platelets) in the bloodstream drops. This can lead to shock, internal bleeding, organ failure, and even death [28]. Inhibiting this will help further prevent heart disease and improve the management of DHF. For that, we identified several effective targeted drugs with our repurposing approach, including aceclofenac, acetaminophen, aspirin, choline magnesium trisalicylate, diclofenac, etodolac, epinephrine, indomethacin, ketoprofen, ketorolac, loxoprofen, mefenamic acid, meloxicam, nabumetone, naproxen, phenyl salicylate, suprofen, and lenalidomide. Controversially, aspirin is an antiplatelet drug that prevents clotting of the blood in dengue patients, and it has been noted that high-dose (>1 gram) aspirin was linked to increased bleeding risk, probably because of its permanent antiplatelet effects [29], making it important to accurately decide the dosage while treating the condition. Despite the wide range of increased procoagulant activity during sickness, thrombotic events have not been extensively recorded in dengue, even though hemorrhagic events of various degrees have often been described [30]. Between January and March 2011, there was a localized dengue fever outbreak brought on by dengue virus type 1 and dengue virus type 2 in Brazil; individuals with dengue fever experienced multiple incidences of thrombotic events that impacted large veins [31]. These cases were similar to others reported in different parts of the world. Enrichment analysis emphasizes that most pathways of the immune system are highly enriched, and adult and immune system–associated tissues were associated with an enriched viral and drug response throughout the study. The network-based analysis suggested that 3 drugs have repurposable properties: aspirin, captopril, and rilonacept; however, because of their anticoagulant qualities, patients should be encouraged to be properly hydrated and avoid aspirin (ie, acetylsalicylic acid), aspirin-containing medicines, and other nonsteroidal anti-inflammatory drugs, such as ibuprofen [30]. On the other hand, several studies have reported that thrombosis is associated with dengue fever [32,33]; hence, further studies are essential to prove the efficiency of aspirin and the other repurposed drugs identified in this study for the treatment of DHF.

### Limitations of This Study

This study took into account all targets of DHF and sorted them based on scores from databases, but the data set represents a large population size, so it may or may not be generalizable to groups within the population. In the real world, the expressed genes may vary based on ethnicity, heredity, and drug use, so it is essential to test the most expressed genes and their associations with the pathogenesis of DHF. A network-based drug reprofiling approach will be helpful to enable effective personalized medicine, but whole-genome sequencing is still not a cost-effective method compared to traditional treatment methods.

### Conclusion

Aspirin is not usually prescribed for dengue fever because of bleeding complications, but it has been reported that using aspirin in lower doses is beneficial in the management of diseases with thrombosis. Drug repurposing is an emerging field in which clinical validation and dosage identification are required before drugs are prescribed. Further retrospective and collaborative international trials are essential for understanding the pathogenesis of this condition.

## Appendix

Multimedia Appendix 1 Functional genes identified from GeneCards and related literature.

Multimedia Appendix 2 Host-DHF interactome.

Multimedia Appendix 3 Results of Kegg pathway analysis and reactome pathway analysis.

Multimedia Appendix 4 Host–DHF–antiviral drug interactome.

Multimedia Appendix 5 Kegg pathway gene enrichment analysis.

Multimedia Appendix 6 Host–DHF interactome–anti–dengue hemorrhagic fever drug interactome.

Multimedia Appendix 7 Most enriched gene pathways according to Kegg pathway analysis.

Multimedia Appendix 8 Higher resolution version of Figure 1. Human-anti–dengue hemorrhagic fever–antiviral drug–anti–dengue hemorrhagic fever interaction network. ADHF: anti–dengue hemorrhagic fever; AV: antiviral; DB: DrugBank; DHF: dengue hemorrhagic fever.

Multimedia Appendix 9 Higher resolution version of Figure 2. Enrichment analysis of human-anti–dengue hemorrhagic fever–antiviral drug–anti–dengue hemorrhagic fever. (A) Kegg pathways, (B), reactome pathways, and (C) tissues.

Multimedia Appendix 10 Higher resolution version of Figure 6. Functional hub gene enrichment analyses: (A) reactome pathways, (B), Kegg pathways, and (C) tissues.

Multimedia Appendix 11 Higher resolution version of Figure 7. Functional hub gene–drug enrichment analyses: (A) hub targets, (B) side effects, and (C) biological process.

## Abbreviations

*ACE*: angiotensin converting enzyme
AGE-RAGE: advanced glycation end products–receptor for advanced glycation end products
CI: credible interval
DHF: dengue hemorrhagic fever
*F2*: coagulation factor II, thrombin
FDA: Food and Drug Administration
HP: host-pathogen
PPI: protein-protein interaction
*PTGS2*: prostaglandin-endoperoxide synthase 2
WHO: World Health Organization
