# Supplementary figures and images for: The Identification of Potential Drugs for Dengue Hemorrhagic Fever: Network-Based Drug Reprofiling Study
Source: JMIR Bioinform Biotechnol. 2023 May 9;4:e37306. doi: 10.2196/37306 (PMC11135182; doi:10.2196/37306)

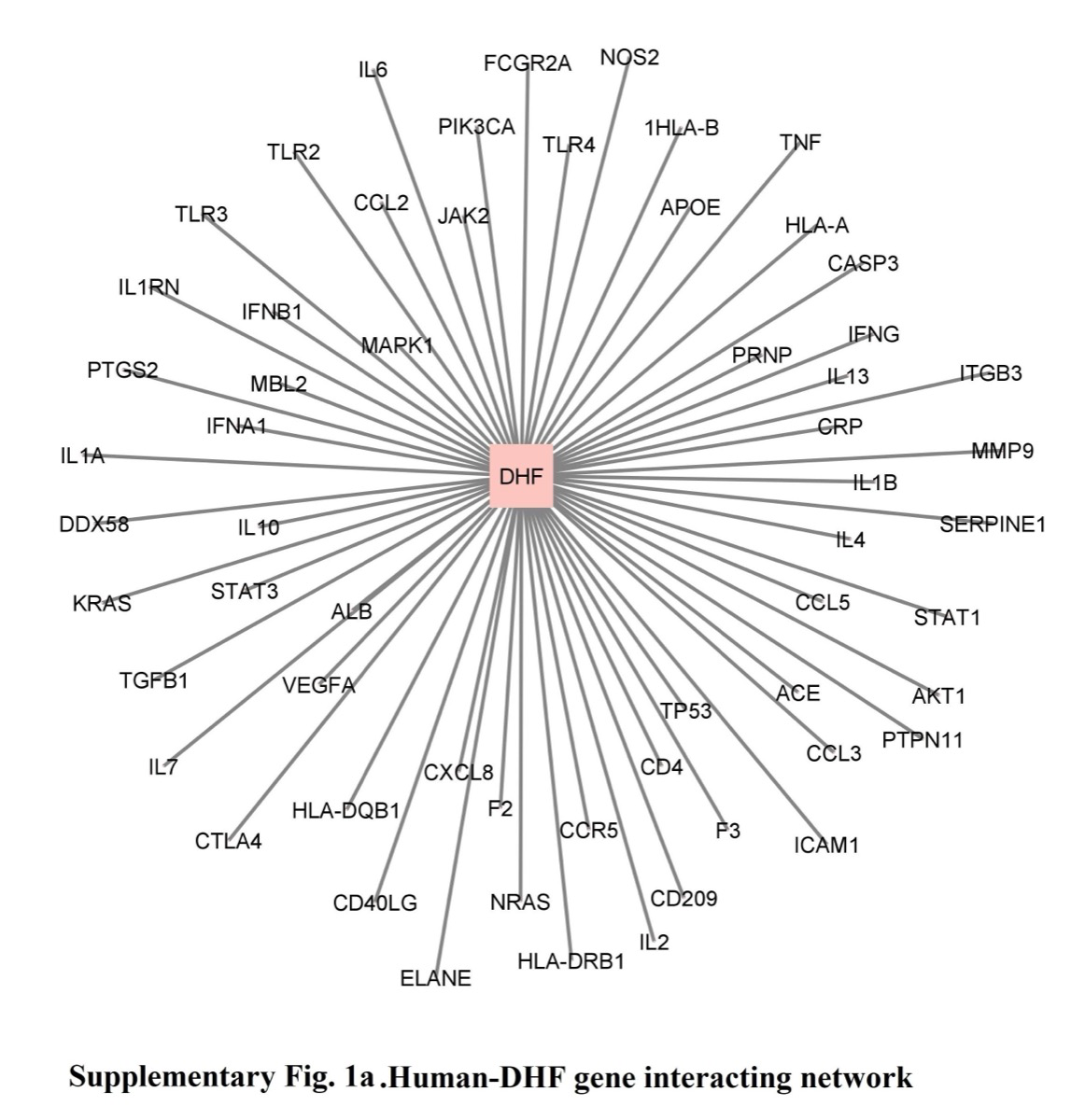

Supplement: Multimedia Appendix 2 [file bioinform_v4i1e37306_app2.png]

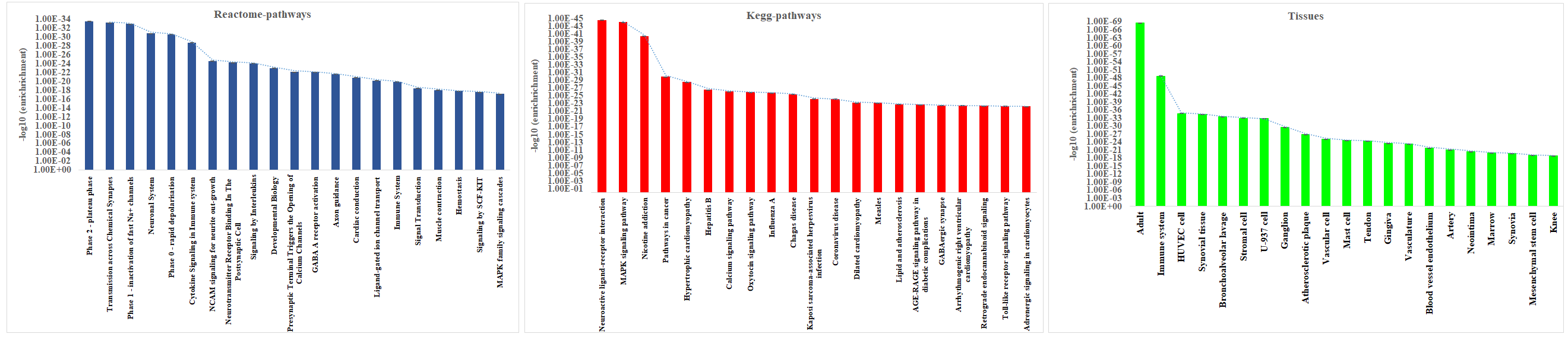

Supplement: Multimedia Appendix 3 [file bioinform_v4i1e37306_app3.png]

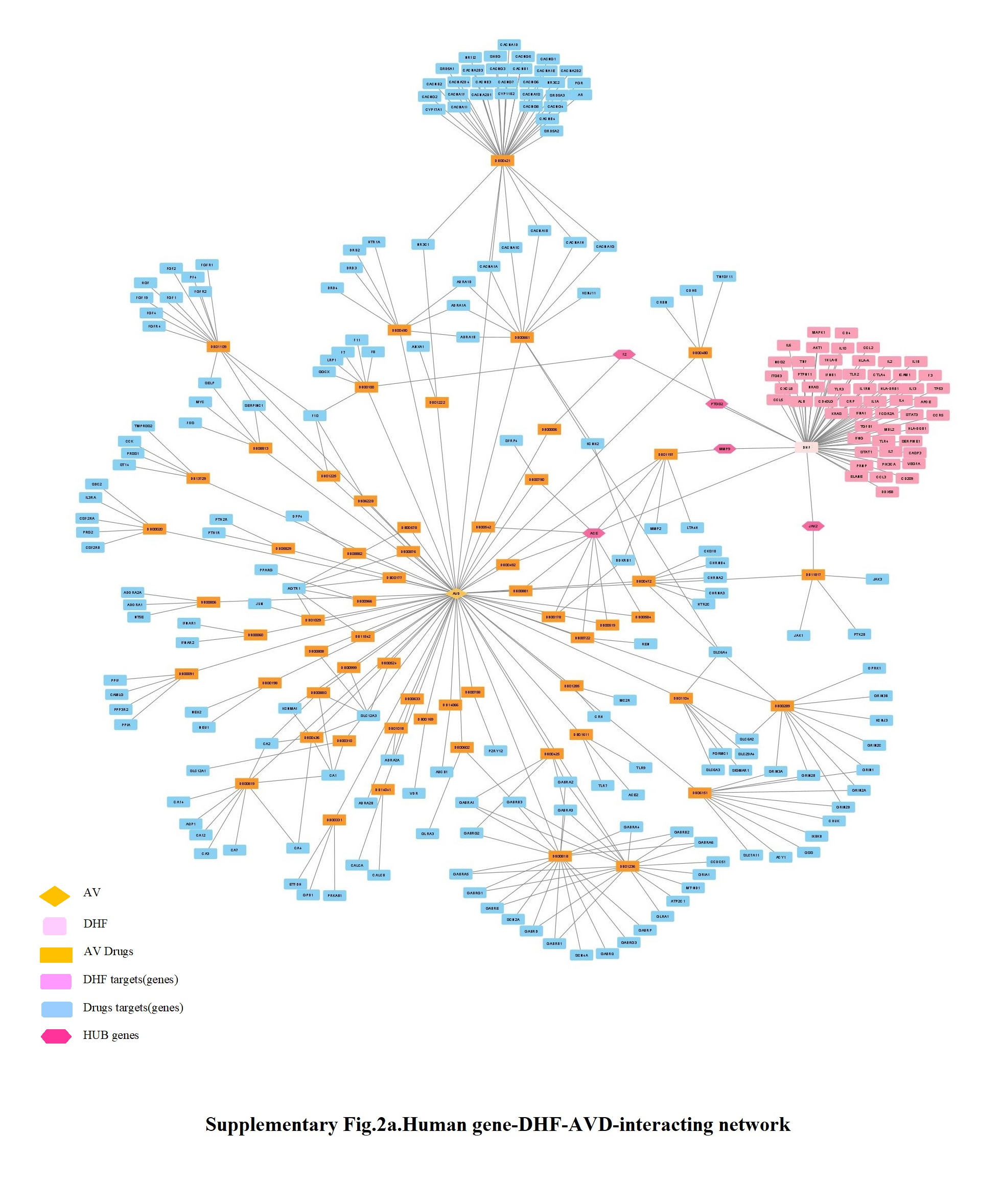

Supplement: Multimedia Appendix 4 [file bioinform_v4i1e37306_app4.png]

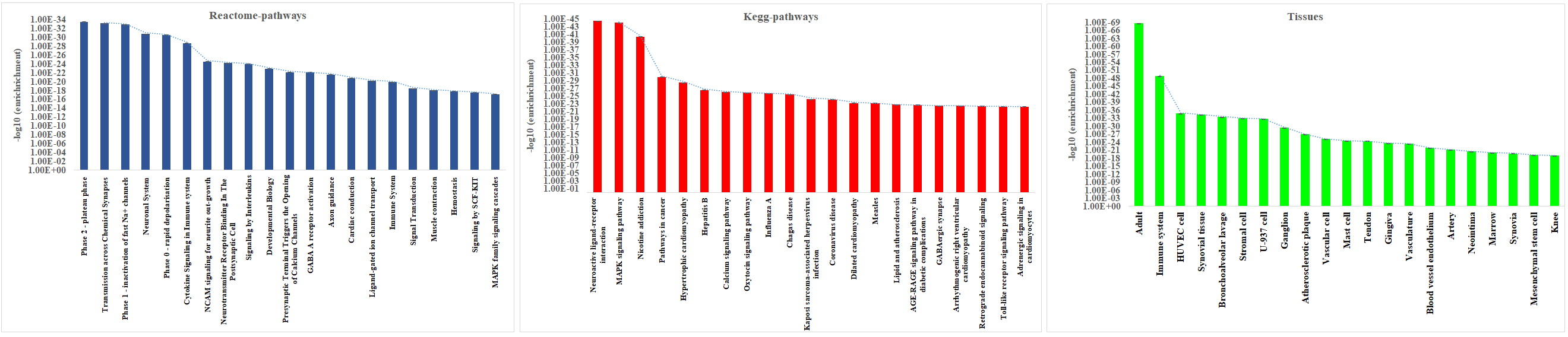

Supplement: Multimedia Appendix 5 [file bioinform_v4i1e37306_app5.png]

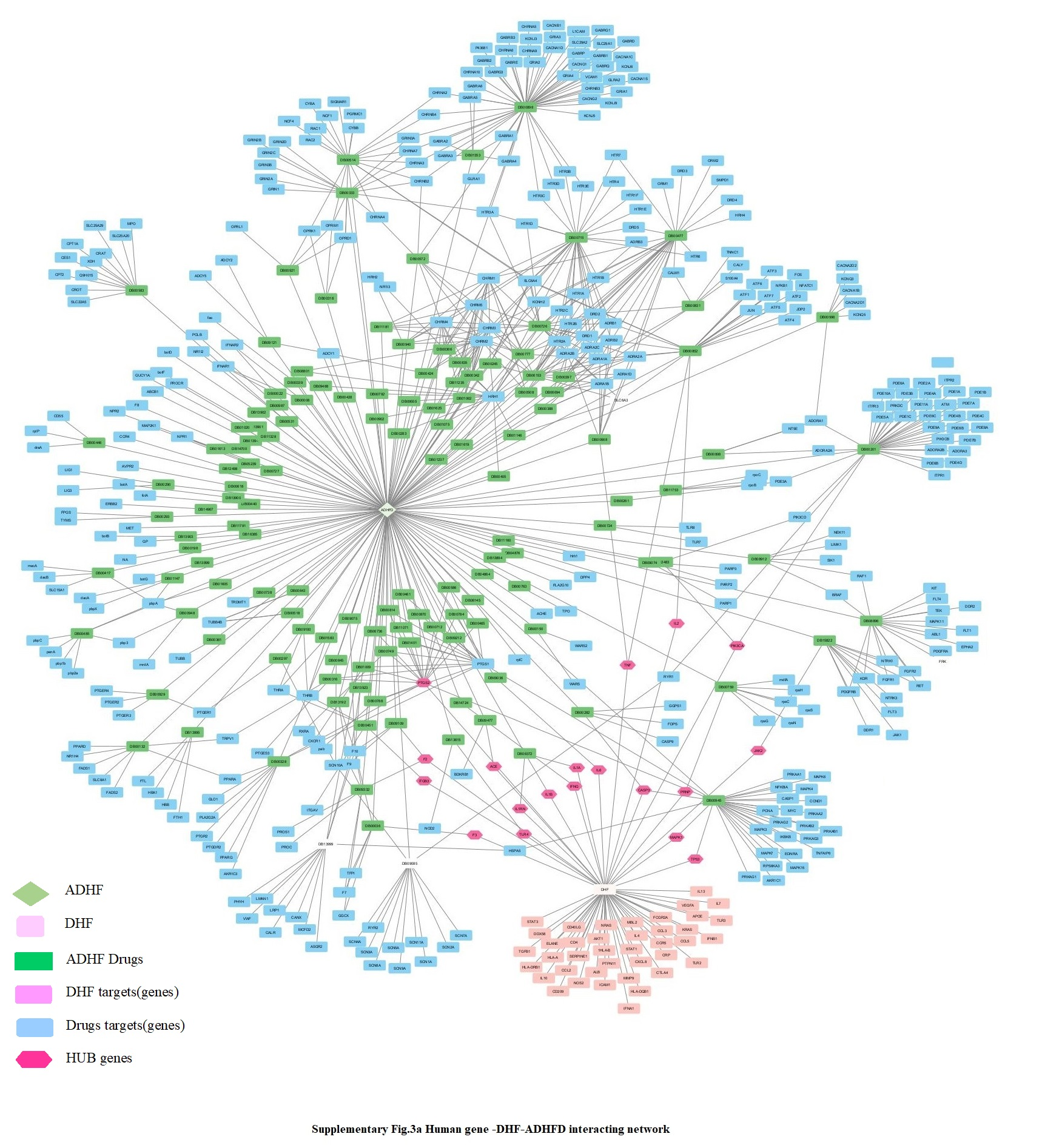

Supplement: Multimedia Appendix 6 [file bioinform_v4i1e37306_app6.png]

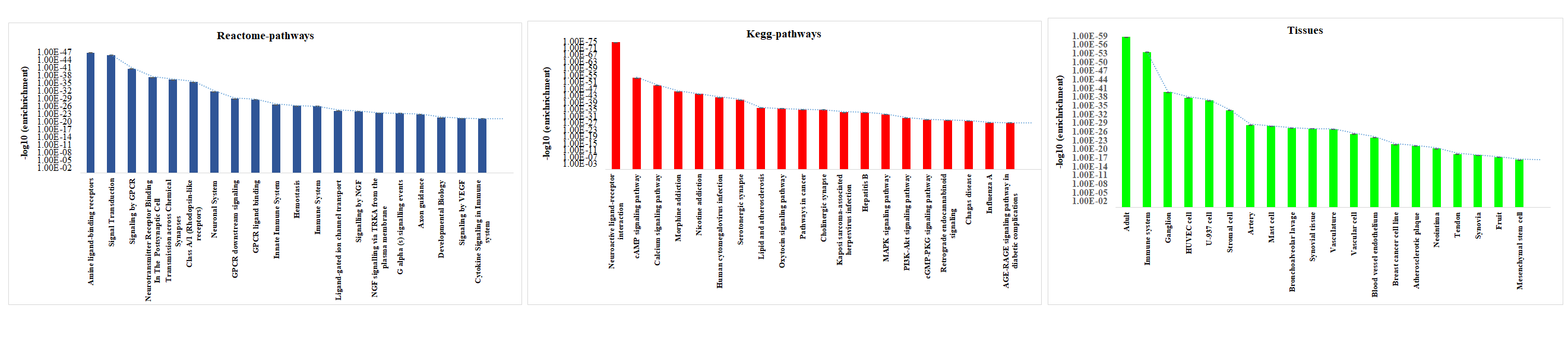

Supplement: Multimedia Appendix 7 [file bioinform_v4i1e37306_app7.png]

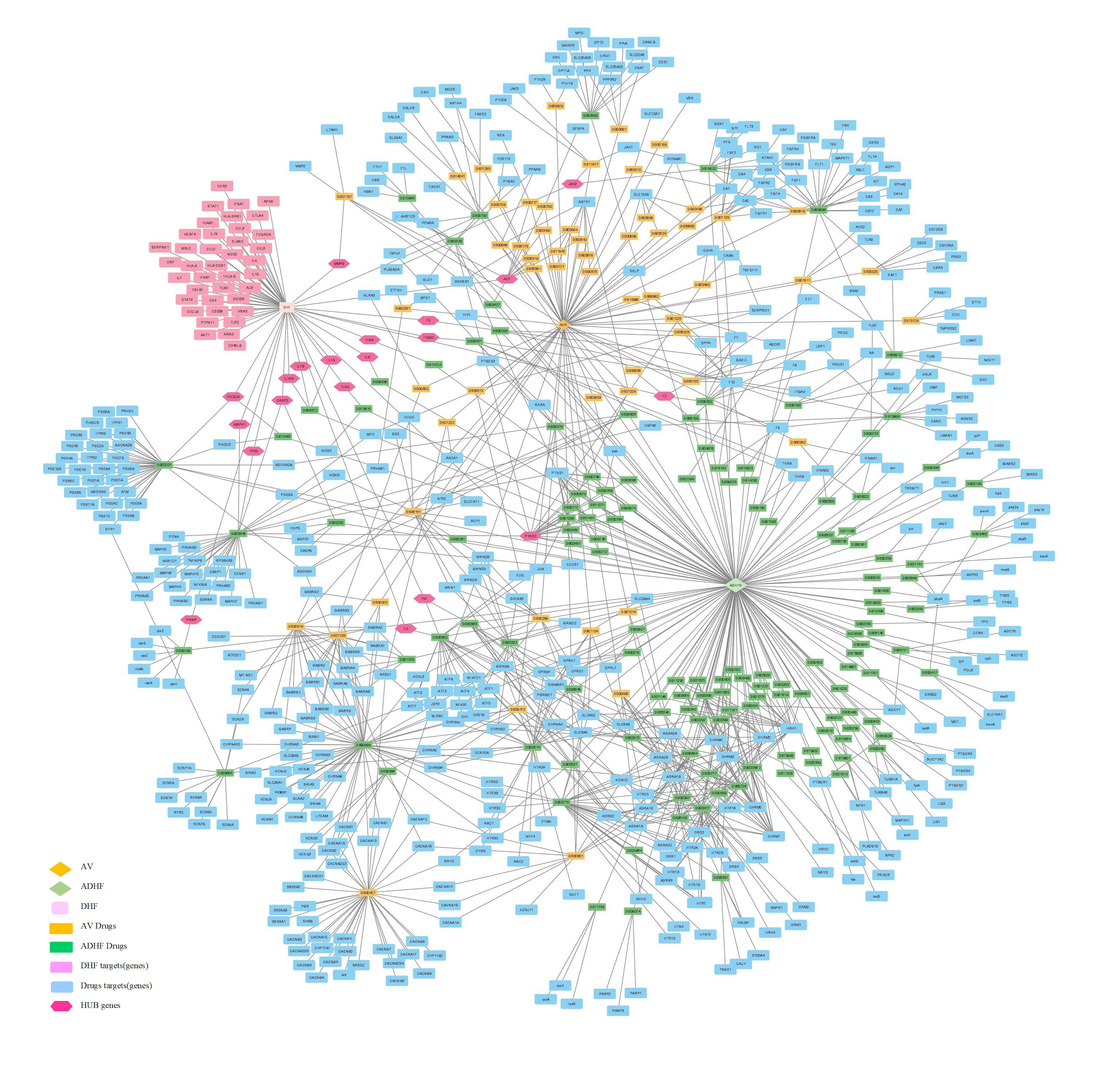

Supplement: Multimedia Appendix 8 [file bioinform_v4i1e37306_app8.png]

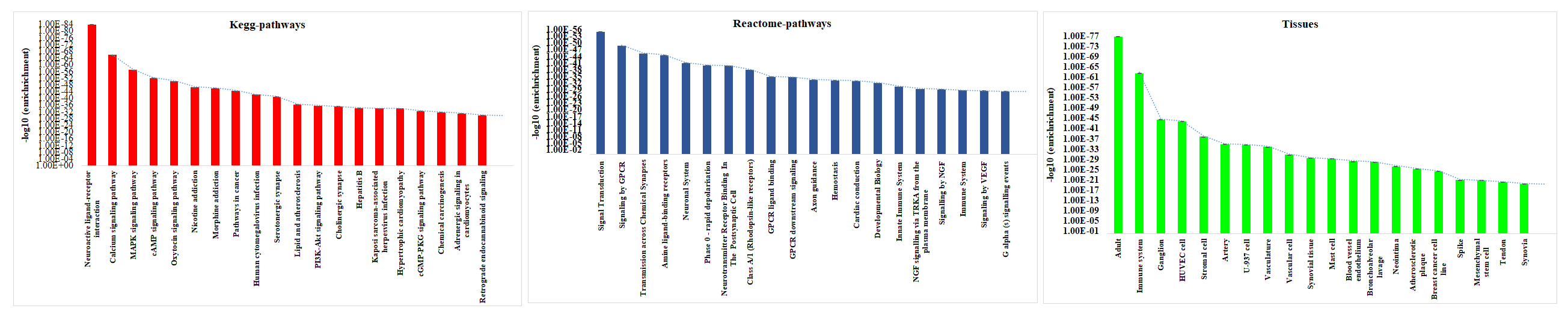

Supplement: Multimedia Appendix 9 [file bioinform_v4i1e37306_app9.png]

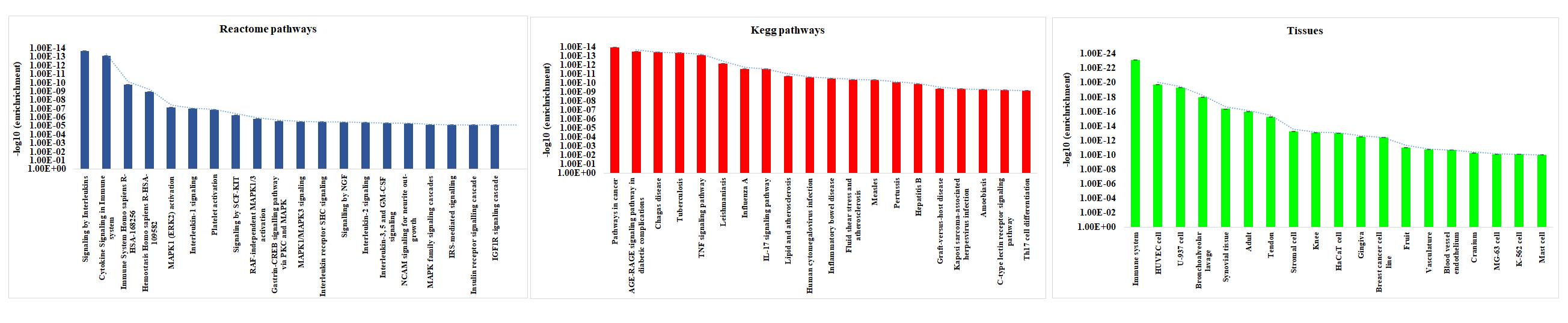

Supplement: Multimedia Appendix 10 [file bioinform_v4i1e37306_app10.png]

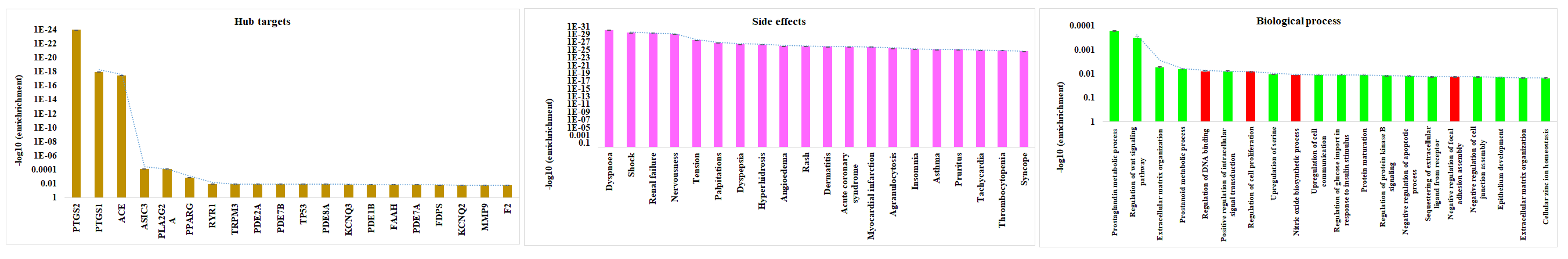

Supplement: Multimedia Appendix 11 [file bioinform_v4i1e37306_app11.png]
